# Habitat associations and impacts on a juvenile fish host by a temperate gnathiid isopod

**DOI:** 10.1016/j.ijppaw.2021.12.006

**Published:** 2021-12-13

**Authors:** Claire A. Spitzer, Todd W. Anderson, Paul C. Sikkel

**Affiliations:** a Department of Biology and Coastal and Marine Institute, San Diego State University, San Diego, CA, 92182, USA; b Department of Marine Biology and Ecology, Rosenstiel School of Marine and Atmospheric Science, 4600 Rickenbacker Causeway, Miami, FL 33149, USA; c Water Research Group, Unit for Environmental Sciences and Management, North-West University, Potchefstroom 2520, South Africa

**Keywords:** Fish, Ecology, Animal behavior, Micropredation, Benthic, Sub-tidal, Gnathiid

## Abstract

The distribution and abundance of organisms is typically shaped by multiple biotic and abiotic processes. Micropredators are parasite-like organisms that are smaller than their hosts and/or prey and feed on multiple hosts during a given life stage. Unlike typical parasites, however, they spend much or most of their time free-living, associating only temporarily with hosts. In the ocean, micropredators can impact multiple fish species, and in particular can have significant lethal and sub-lethal effects on newly settled fish. Although gnathiid isopods are abundant and primary micropredators in coral reef ecosystems, their impacts are relatively unexplored within sub-tidal temperate rocky reefs. We investigated the distribution of juvenile gnathiid isopods along sub-tidal temperate rocky reefs and tested trap methodology. We also quantified both the sub-lethal and lethal impacts of feeding-stage juvenile gnathiid isopods on juvenile, post-settlement reef fish, *Heterostichus rostratus* (giant kelpfish). We were most interested in determining the relationship between gnathiid infestation level and fish swimming performance, in particular swimming metrics relevant to predator avoidance maneuvers. We found that *Gnathia tridens* was present in rocky reefs rather than embayments along the Southern California coastline and that within rocky reefs, gnathiids occurred in the highest densities in lighted traps. Surprisingly, we observed almost no influence of fish size or gnathiid sub-lethal infestation level on ambient or burst swimming performance metrics. However, burst duration was reduced by gnathiid infestation, which is important in predator avoidance. There were significant differences in survivorship among small fish compared to large fish as a result of gnathiid infestation. Larger fish survived higher numbers of gnathiids than smaller fish, indicating that parasite-induced mortality is greater for smaller fish. Investigations of the effects of micropredators on subsequent predator-mediated mortality, including the susceptibility of fishes and their individual responses to micropredators, can further contribute to our understanding of processes affecting recruitment in resident reef fish populations. Further research, especially within temperate sub-tidal ecosystems, is needed to understand and highlight the overlooked importance of micropredation in shaping fish populations within a reefscape.

## Highlights

• Gnathiid isopods are more abundant in sub-tidal rocky reef than in bay habitats off southern California.
• Among the trap designs tested, lighted traps were most effective.
• Wave height and lunar period also impacted capture rates.
• For *H. rostratus*, gnathiids alter fish swimming performance at varying levels of infestation.
• Mortality rates from gnathiid infestation were inversely related to the size of fish host.

## Graphical abstract

**Figure:**
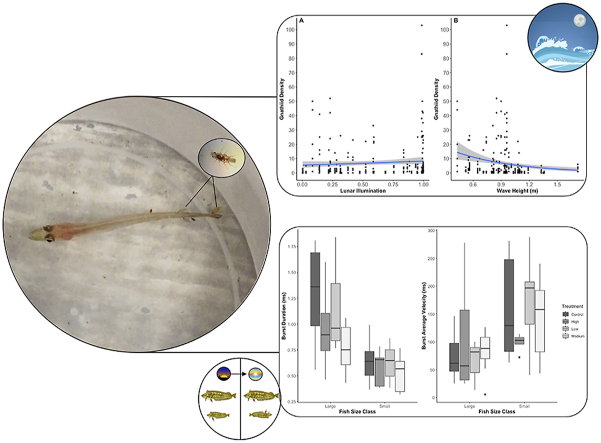

## 1 Introduction

Parasitism is the most common animal lifestyle, and thus most common biological interaction, evolving multiple times and in nearly every animal taxon (e.g. Poulin, 2014; Smit et al., 2019). As a feeding guild, parasites make up ∼40% of global biodiversity (Hatcher and Dunn, 2011). Parasite biomass rivals that of top predators (Kuris et al., 2008), and because of parasites’ effects on host population dynamics, they have a compounding direct and indirect influence on the biodiversity of ecological communities (Mouritsen and Poulin, 2005).

The parasitic Crustacea constitute over 14,000 species from 400 genera that feed mainly or exclusively on vertebrate blood and body fluids (Gracasouza et al., 2006). Isopod crustaceans are among the largest and most diverse orders with 89% of members inhabiting marine environments (Kensley, 1998). Of these, two families (Gnathiidae and Aegidae) are exclusively temporary parasites of fishes, one partially so (Corallanidae), and one a permanent parasite (Cymothoidae).

Among the parasitic isopods, gnathiids are potentially the most important ecologically**,** having even been included among priority “species” to support the functional integrity of coral reefs (Wolfe et al., 2020). Gnathiids are found in temperate, polar, and tropical oceans, from tide pools to the deep ocean (Smit and Davies, 2004; Tanaka, 2007; Quattrini and Demopoulos, 2016). They differ from other marine ectoparasites in two fundamental ways. First, they are only parasitic during each of three juvenile phases (instars), and adults do not feed. Second, with possibly the exception of species that infest sharks and rays, they associate only temporarily with hosts, spending most of their life in the substratum. Thus, they have been referred to variously as “temporary ectoparasites”, “protelean parasites”, and “micropredators”. First-stage parasitic juvenile gnathiids emerge from the substratum, feed on a single host fish and, and when engorged on blood or body fluids, return to the substratum and molt into the next stage. This cycle is repeated a second and third time (Smit and Davies, 2004), with each feeding event on a different host (Smit and Davies, 2004). The unfed, mobile stages are “zuphea” juveniles, and juveniles that have ingested a blood meal are “pranizae” juveniles. After the final blood meal, third-stage juveniles metamorphose into non-feeding adults that live in the benthos, reproduce, and then die. Females retain eggs in a brood pouch (marsupium) until hatching of post-embryonic first-stage juveniles (Manship et al., 2012). Gnathiids rarely swim more than a meter above the substratum (Nicholson et al., 2020) and have no pelagic dispersal phase. In tropical species, the life cycle is completed in about a month (*Gnathia marleyi*; Sikkel and Welicky, 2019), but in polar regions can take over two years (Wägele, 1988).

Through their blood meals, gnathiids can have multiple impacts on hosts. Physiologically they can cause reduced hematocrit (Jones and Grutter, 2005), increased levels of corticosteroid stress hormones (Triki et al., 2016), impaired cognitive function (Binning et al., 2018), and the creation of wounds that can lead to secondary infection (Honma and Chiba, 1991). In cases of extreme infestation, they can cause death in adult hosts (Mugridge and Stallybras, 1983; Hayes et al., 2011). However, for juvenile fishes, even a single gnathiid can prove fatal (Grutter et al., 2008, 2017; Artim et al., 2015; Sellers et al., 2019), and sub-lethal infestation can impact performance (Sellers et al., 2019; Allan et al., 2020).

The temporary nature of interactions with hosts and their small size have rendered gnathiids largely unnoticed by marine ecologists. Moreover, the vast majority of work on their habitat associations and impacts on hosts have been conducted in coral reef environments, whereas little has been done in temperate ecosystems (Sikkel and Welicky, 2019). Our goals were twofold: (1) assess the spatial patterns of abundance of gnathiid isopods within the warm-temperate sub-tidal habitats of southern California, including rocky reefs and soft-bottom embayments, and (2) assess the size-dependent impacts of gnathiids on mortality and swimming performance of juveniles of a common coastal reef fish, the giant kelpfish, *Heterostichus rostratus* (Clinidae) that is found in both habitats.

## 2 Methods

### 2.1 Densities of gnathiids in rocky reefs vs. soft-bottom embayments and a comparison of trap effectiveness

#### 2.1.1 Field methods

From April–October 2016, trapping studies were conducted at two rocky reefs (Bird Rock (32°48′36 N, 117°16′36 W) and Sunset Cliffs (32°43′51 N, 117°15′46 W)) and in two embayments, Mission Bay (32°46′222 N, 117°14′543 W) and San Diego Bay (32°42′44 N, 117°13′37 W)) off San Diego, California, USA. Emergence traps were deployed using SCUBA to determine the densities of gnathiid isopods within rocky reefs and embayments. Three trap types were used to determine which type captured the most gnathiids. To compare trap effectiveness, a subset of traps were modified to include a live fish (“fish-baited’) or submersible light (“light-baited”; see below for details). Within each site, one of each trap type was randomly deployed (“fish”, “light”, “control”) within two different types of habitat for rocky reefs (rock and sand) and for embayments (sand and seagrass). All benthic emergence traps were deployed at 0900 for 24 h. At the end of the 24 h sampling period, the bottle was removed from a trap and the rubber stopper was pushed into the funnel, sealing the sample. Samples were placed on ice and transported to the San Diego State University (SDSU) Coastal and Marine Institute Laboratory (CMIL) where all gnathiids were counted. Samples were placed on ice to prevent samples from experiencing elevated temperatures during transport from field sites to laboratory. All samples were sorted alive, both zuphea and pranizae stage gnathiids were noted and counted.

**Fig. 1:**
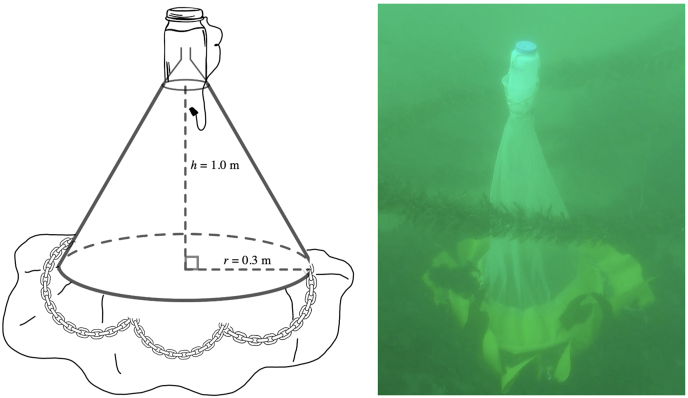
Design schematic and photograph of *in situ* emergence trap used for sampling. A Control trap is illustrated. “Light-baited” and “Fish-baited” traps used the same design but contained a single submersible light (for light-baited) or a 60–90 mm giant kelpfish (for fish-baited) within the 1 L plastic bottle at the top of the traps.

*Trap design*: Modified benthic emergence traps followed the designs of Grutter et al. (2000) and Chambers and Sikkel (2002) (Fig. 1), so that densities per unit area could be obtained. Traps were made of 100 μm nylon mesh that formed a pyramidal cone, with a 0.6-m diameter plastic hoop base enclosed by a 20-cm impermeable coated nylon skirt that surrounded the nylon mesh collecting cone. An inverted plastic funnel was soldered to a 1-L plastic bottle filled with seawater that was attached to the mesh cone. Each bottle was filled with 800 ml of seawater and affixed to a plastic fitting on the nylon mesh cone, resulting in a buoyant cod end. Funnels within each collection bottle were closed with rubber stoppers to ensure that each sample was sealed during trap deployment. Gnathiids emerged from the substratum and entered the collection bottle through the funnel. To prevent openings between the trap base and any uneven substratum, the nylon skirt was weighted to the sea floor with 7 m of galvanized chain surrounding the circumference of each trap, which allowed us to quantify the density of gnathiids per trap (trap area sampled = 0.28 m^2^).

*Trap modifications:* “Lighted traps” received a single rechargeable dive light rated at 90 lumens (Princeton Tec AMP 1.0 Dive Light, Trenton, New Jersey, USA), which was placed within the cavity of the 1-L plastic bottle, above the funnel. Control traps consisted of the trap only without fish or a light. “Fish-baited” traps were baited with giant kelpfish that ranged in size from 60 to 90 mm total length (TL). Live giant kelpfish were chosen as bait because this species was common in both embayments and rocky reefs. Fish deployed in traps were captured as needed by beach seine from eelgrass beds at Shelter Island, San Diego Bay (32°42′43 N, 117°13′39 W) and Ventura Cove, Mission Bay (32°46′16 N, 117°14′40 W). All fish were collected under California Department of Fish and Wildlife collection permit SC-13350 and fish were handled using the approved SDSU IACUC protocol APF 15-10-013A. Fish were transferred from the seine into an aerated insulated plastic container and transported to CMIL within 30 min of capture. Fish were transferred to holding aquaria (75 cm long x 30 cm wide x 30 cm high; 75 L volume) in a flow-through seawater system. All fish were provided with a 25-cm^2^ ASU (artificial seagrass unit; 50 shoots) for refuge. Fish were fed once a day with live grass shrimp (*Hippolyte californiensis*) and transferred to an aerated insulated container prior to being placed into a trap on a given day. Fish were fed until satiation prior to being transferred to benthic emergence trap containers. *Heterostichus rostratus* do not reach sexual maturity until adulthood (1–2 years of age) at which point they can be identified by gender, giant kelpfish used in this trapping study were juveniles and thus could not be sexed (Stepien, 1986). Each fish was used only once and never reused in subsequent studies.

Based on the results from our 2016 trapping study, we chose to further assess the densities of gnathiids within rocky reefs only because of clear differences in the numbers of gnathiids collected in rocky reefs versus those in embayments (see Results). Our goal was to investigate abiotic factors, in particular lunar illumination (a decimal proportion (0–1, 1 being 100% full moon illumination), wave height (m), wave period (s), and sea surface temperature (SST) (C^o^), that may influence gnathiid densities within rocky reefs. Sampling for this study was conducted over rocky reefs spanning 13 km between La Jolla (32°48′54 N, 117°17′20 W) and Point Loma (32°42′14 N, 117°16′5 W), San Diego, California, USA. This sampling area consists of large continuous rocky reefs, kelp forests, and sand patches. The substratum within this area is a matrix of rocky reef covered with understory algae interspersed with sand channels. This area was chosen for its' mosaic patchwork of rocky reef and sandy bottom substrata.

We deployed lighted emergence traps because they were far more effective at collecting gnathiids than fish-baited or control traps (see Results). We deployed these traps biweekly using SCUBA for a 1-yr period, from Apr (2017) through Apr 2018. For each sampling period, six traps were placed haphazardly (three in sand substrate and three on rocky reef matrix) at depths of 10–12 m. Traps were deployed at 0900 for 24 h at one site, retrieved the following day, and immediately reset at the other location, totaling 3 successive days, with the order of the sites for each sampling period chosen randomly. Samples were transported to the laboratory where all gnathiids were sorted and counted. Due to adverse weather conditions, no sampling occurred in January 2018.

#### 2.1.2 Data analysis

Gnathiid densities were compared between rocky reefs and embayments with a Mann-Whitney *U* test because data did not meet assumptions of normality. Because of clear differences in the numbers of gnathiids collected in rocky reefs versus those in embayments, we conducted all subsequent sampling and analyses at rocky reef sites. All statistical analyses were performed using R software vs. 4.0.4 (R Core Team, 2021).

To assess the efficacy of our traps in collecting gnathiids, we analyzed differences in gnathiid density between trap types (lighted, fish-baited, control), using a generalized linear model with a negative binomial error distribution to account for overdispersion. We assessed other potential distributions by looking at model fit and assumptions. Trap type was the sole categorical fixed factor in the model. We used the function “glm.nb” from the package “MASS” (Venables and Ripley, 2002) and the function “Anova” from the package “Car” (Fox and Weisberg, 2011). Post-hoc multiple comparisons were made using the package “emmeans” and applying Tukey corrections (Lenth 2018).

Additionally, we analyzed for differences in gnathiid density using a negative binomial generalized linear model (GLM), with negative binomial error distribution (log-link function) to account for overdispersion. Substratum type (sand, rock) was a fixed effect, lunar illumination (a decimal proportion (0–1, 1 being 100% full moon illumination), wave height (m), wave period (s), and sea surface temperature (SST) (C^o^) were continuous factors, and gnathiid density was the response variable. The negative binomial GLM analysis was conducted using the “glm.nb” function in the R package “MASS” (Venables and Ripley, 2002). Counts are often over-dispersed and zero-inflated (Yau et al., 2003); thus we used generalized linear mixed modeling (GLM) to analyze our data. Additionally, we chose GLM's due to their ability to accommodate variance heterogeneity and non-normal distributions (Venables and Dichmont, 2004). The full model, with all possible interactions, was tested, and a final simplified model was selected based on the corrected Akaike information criteria (AICc). We chose to use AICc due to the small sample size of the dataset (Burnham and Anderson, 2002, 2004; Johnson and Omland, 2004). We ensured that models fit assumptions using model diagnostics. AICc values and all potential model outputs were created using the “dredge” function in the “MuMIn” package. We selected models using AICc with the “MuMIn” package and performed all model diagnostics in the “DHARMa” package. We selected the model with the lowest AICc (i.e., AIC difference higher than 2, most parsimonious model) (Symonds and Moussalli, 2011). We confirmed that models fit assumptions using model diagnostics. For the overall model, we calculated a pseudo-R^2^ in the “sjmisc” package.

### 2.2 Gnathiid impacts on juvenile fish

#### 2.2.1 Collection of fish and gnathiids

Gnathiids were collected from rocky reefs (10–15 m depth) at Point Loma and La Jolla, California, USA using small illuminated bottle traps (modified from the design of Jones and Grutter, 2007), with each bottle weighted with a brick and suspended 0.5 m above the benthos. Traps were set at 1400 and collected at 0900 the following day. Bottles were sorted and gnathiids removed by pipette and transferred into 3.75 L holding tanks containing seawater. Juvenile gnathiids (zuphea) were kept in holding tanks for 1–3 d before their use in experiments. Only unfed gnathiids, all similar in size, were used in experiments. Unfed zuphea were used because they are the only feeding stages within the gnathiid life cycle, which was necessary for infestation experimentation. Infestation of experimental fish was carried out between 1630 and 1930.

Larger (42–50 mm standard length (SL) and smaller (24–30 mm SL) juvenile giant kelpfish were captured by beach seine from eelgrass beds at Shelter Island (San Diego Bay) and Ventura Cove (Mission Bay) between May 2016 and Sep 2017 and transferred to CMIL. All fish were examined for additional external parasites, and those deemed “unhealthy” were removed. “Unhealthy” was characterized as any apparent injury or infection externally or on the gills, or any apparent malnutrition.

#### 2.2.2 Laboratory experiments

Prior to swimming trials, four kelpfish from a single size category (small or large juveniles) were haphazardly selected and removed from holding aquaria and placed into four individual cylindrical plastic containers (250 ml polypropylene wide mouth container, DWK Life Sciences-Wheaton, Millville, New Jersey, USA) in a separate room. A black divider (1 m^2^) was placed between aquaria so that fish could not observe one another. No other stimuli were present in the experimental room. Fish in each container were randomly assigned to one of four treatments in a given trial: (1) a control with a fish exposed to no gnathiids, (2) a fish exposed to three gnathiids (low level), (3) a fish exposed to six gnathiids (medium level), and (4) a fish exposed to nine gnathiids (high level). Gnathiid exposure levels were determined by preliminary trials, which identified lethal levels of gnathiid exposure on juvenile kelpfish, in addition to scaled results by fish length (Grutter et al., 2017; Artim et al., 2015; Sellers et al., 2019). Gnathiids and fish used in a trial were replaced with other gnathiids and fish for each subsequent trial. For each size category of fish, there were seven replicates per treatment.

Gnathiids for each treatment were added to a cylindrical plastic container one at a time using a 5-ml pipette. Gnathiids were allowed to feed for 14 h, determined by duration of night-time hours and preliminary experiments aimed at assessing mean feeding times of gnathiids. After this time period, the number of gnathiids that had fed, were currently feeding, and had not fed were recorded as well as fish status (dead or alive). Living fish were then moved to aquaria (40 cm long x 20 cm wide x 26 cm high; 18L capacity) with flow-through seawater and filmed. Fish were sequentially moved one individual at a time into a filming aquarium by placing the cylindrical plastic container inside the aquaria so that fish could swim freely into the aquarium from the container without handling.

Fish were acclimated in each aquarium for 20 min. The timing of initial gnathiid infection was staggered for each treatment to accommodate filming a single treatment at a time while maintaining a 14 h infestation period, with the goal of filming fish from all four treatments in the morning on a single day. Water height in filming was maintained at 22 cm for all trials. Fish were filmed in all trials using two GoPro cameras (GoPro Hero 4, GoPro Inc., San Mateo, CA, USA), one directly facing the aquarium and one directly facing down from the top of the aquarium to provide estimates of both vertical and horizontal swimming, respectively. In cases where gnathiids disappeared (presumed to have been eaten by the fish), the gnathiids or fish had died, or the gnathiids had not fed in time for the fish to be tested, the fish was not used for performance trials (n = 15 aborted trials).

At the beginning of each acclimation period, both cameras were turned on (not actively filming); after the acclimation period, the cameras were remotely switched to film mode and subsequently filmed in each aquarium for 20 min at 60 frames • s^−1^. To measure fish movement, we used the MtrackJ plugin in ImageJ v 2.0 software (Schneider et al., 2012). For both overhead and side-view cameras, we tracked the two-dimensional coordinates of the fish's anterior tip of the snout as it moved through the filming aquaria. During the first 10 min of a trial, ambient swimming of a fish was recorded. Fish were tracked every 100 frames (∼roughly 300 tracked points per 10 min period). We measured cumulative distance traveled (distance in mm), and mean velocity while traveling (mm/s). Following this 10 min period, fish were stimulated by thrusting a glass probe in the water behind each fish to induce a burst swimming response (Batty and Blaxter, 1992; Billerbeck et al., 2001; Fisher et al., 2007); two stimulus events were completed during this 10-min period. After the stimulus was applied, we tracked a fish's movement frame-by-frame for the entire duration of the response until the fish ceased moving for 10 consecutive frames (see Marras et al., 2011). For each burst response, we measured cumulative distance traveled (distance in mm), mean velocity while traveling (mm/s), maximum velocity reached while traveling (mm/s), duration of the burst response (ms), and response latency (time between stimulus onset and fish response in ms). We were only able to analyze each burst response via side video cameras as the overhead view was obstructed by water refraction and the physical stimulus. After each trial, fish were measured (SL) and returned to a separate holding tank where they were then euthanized because they could not be returned to natural settings because of California Department of Fish and Wildlife guidelines.

#### 2.2.3 Data analysis

We assessed how fish size category (small and large) and gnathiid treatment level (control, low, medium, high) impacted a series of fish swimming response variables associated with ambient and burst swimming. Response variables, including cumulative distance traveled (mm), mean velocity while traveling (mm/s), response latency (ms), duration of the burst response (ms), mean burst velocity (mm/s), maximum velocity reached during the burst response (mm/s), and cumulative distance traveled during the burst response (mm) were tested using multivariate analysis of variance (MANOVA, Allan et al., 2020; Friedrich et al., 2018) with fish size category (small and large) and gnathiid treatment level (control, low, medium, high) as fixed factors, and the interaction between treatment and size class. Residual plots were examined to inform MANOVA assumptions of multivariate homogeneity and normality. Wilk's Lamba was used to determine the significance of the overall MANOVA, and was interpreted as the proportion of the variance explained by the model (Todorov and Filzmoser, 2010). Significant MANOVA effects were further examined using ANOVAs and Tukey's HSD post hoc tests, using the “Anova” function from the “Car” package.

Using the binomial density function (Öhman et al., 1998), we tested whether there was a difference in the probability of survival between fish size categories (large and small) at a specific gnathiid treatment level.

Binomial Density Function

$$\text{f}\left(\text{x}\right)\;=\;\text{n!/}\left[\text{x!}\left(\text{n}-\text{x}\right)\text{!}\right]\;{\text{p}}^{\text{x}}{\text{q}}^{\text{n}-\text{x}}$$

The binomial density function used the total number of individuals in a size category for a particular treatment (n), the count of dead fish in that size category (x), the probability of survival in the opposing size category (p; 1 - no. dead individuals/total individuals in that size category), and the probability of death in the opposing size category (q). This function allowed us to analyze whether the probability of survival in the large size category was likely to be the same as the small size category given the counts of alive/dead individuals in the small size category. We determined the likelihood that the small size category counts of alive and dead fish at a particular treatment level departed from the probability of survival in the large size category. We then performed this analysis on both the medium and high gnathiid treatments only as there was 100% survival in the control and low treatments. When calculating the binomial density function, we used the probabilities found in the large size category for each treatment rather than the small size category because the probability of survival for the large size category did not deviate largely between treatments.

## 3 Results

### 3.1 Species identification

**Fig. 2:**
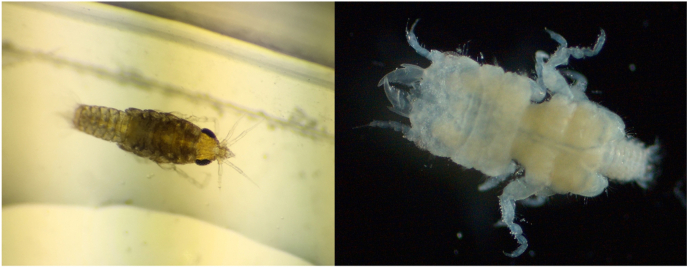
Photograph of a juvenile (left) and adult male (right) *Gnathia tridens* collected during sampling. Adult male specimen was used for species identification (adult male photo and species identification was completed by Nico J. Smit at North-West University).

From our study, the gnathiids collected matched descriptions for *Gnathia tridens* (Menzies and Barnard, 1959) (Fig. 2). Adult males were obtained from third-stage pranizae that were allowed to metamorphose in the lab and sent to taxonomic specialist Nico J. Smit for identification. Specimens are deposited at the Los Angeles County Museum of Natural History.

### 3.2 Densities of gnathiids in rocky reefs vs. soft-bottom embayments and a comparison of trap effectiveness

Gnathiid densities between rocky reefs and embayments were different from one another (Mann-Whitney test, *U* 223, *p* < 0.001). The mean number of gnathiids (±SE) in rocky reef habitat (26.8 ± 15.8) was significantly higher than in embayments (0.0286 ± 0.0286).

**Fig. 3:**
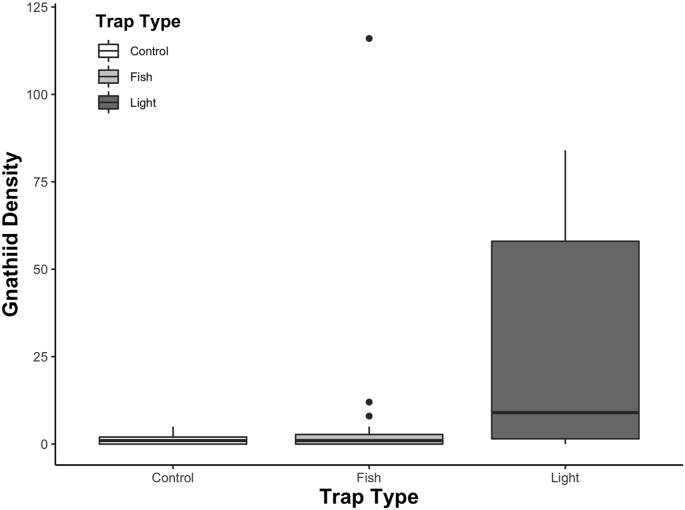
Box plots of gnathiid density (per trap) by trap type. Points represent outliers (>1.5x and <3x of the interquartile range beyond the end of the box; the maximum number of gnathiids collected in a light-baited sample (763) is not shown).

Within rocky reefs, gnathiid density differed significantly among trap types (GLM [negative binomial]: χ^2^ = 32.14, *df* = 2, *p* < 0.001; Fig. 3). Lighted traps overall collected 10 times as many gnathiids compared to fish-baited traps and over 42 times more than control traps. On average, lighted traps collected 42.1 ± 28.2 (±SE), gnathiids per trap compared to fish baited (5.52 ± 4.28) and control traps (0.9 ± 0.260).

**Fig. 4:**
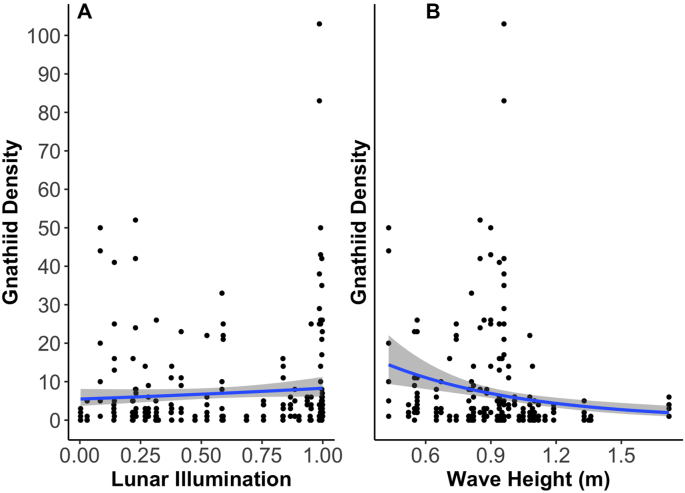
Significant abiotic parameters (lunar illumination and wave height) vs. gnathiid density per trap. Lines represent negative binomial linear regressions with standard error margins.

Overall, our light-baited traps collected a total of 1,670 gnathiids (total of both zuphea and pranizae life stages), with an average density of 7.07 ± 0.854 (±SE) per trap. Gnathiid densities were significantly influenced by lunar illumination (χ^2^ = 5.826, *df* = 1, *p* = 0.015) and wave height (χ^2^ = 15.446, *df* = 1, *p* < 0.001), with a decrease in gnathiid densities as wave height increased and lunar illumination decreased (Fig. 4).

### 3.3 Gnathiid impacts on juvenile fish

**Fig. 5:**
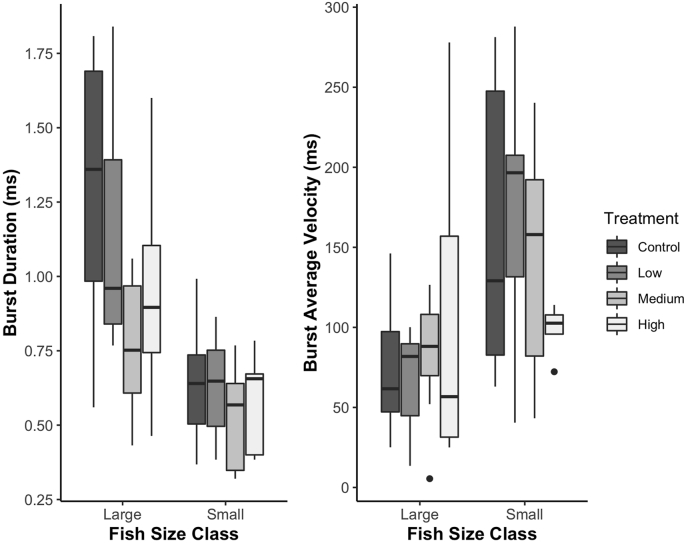
Box plots of significant burst swimming performance metrics by fish size class and gnathiid treatment level. Points represent outliers (>1.5x and <3x of the interquartile range beyond the ends of boxes).

Gnathiid treatment and fish size category did not affect ambient swimming performance metrics (total distance moved (mm) or average velocity while moving (mm/s)), but they did affect the two burst swimming performance metrics (Table 1, Fig. 5). Overall, a significant proportion of the variance in the model was explained by the factor fish size category (Wilks Λ, *p* = 0.0002). Fish size category significantly impacted the mean velocity of a fish's burst response (ANOVA, *F*_1,46_ = 11.835, *p* = 0.001), where smaller fish exhibited faster velocities (mean ± SE) (147 ± 15) than larger fish (82 ± 11). Additionally, the duration of the burst response was impacted by treatment (ANOVA, *F*_3,46_ = 2.915, *p* = 0.044) and fish size category (ANOVA, *F*_1,46_ = 28.125, *p* < 0.001). Larger fish, albeit at a slower average velocity, were able to maintain the burst response for a longer duration of time (large fish = 1.04 ± 0.079, small fish = (0.59 ± 0.036). These results indicate that during a burst response, smaller fish are able to maintain a faster average speed than larger fish but are unable to maintain that response for a longer period of time than larger fish.

**Table 1:** Results of MANOVAs for all swimming performance metrics MANOVAs. The F-statistic is Wilk's Λ, df = degrees of freedom, and significant *p*-values are bolded.

Swimming Metric	*F*	*df*	*P*
Total Distance Traveled (mm)			
Size Class	0.0913	1	0.7639
Treatment	0.9456	3	0.4264
Size Class x Treatment	0.7341	3	0.537
Ave. Velocity (mm/s)			
Size Class	0.8689	1	0.3561
Treatment	2.239	3	0.0963
Size Class x Treatment	1.9528	3	0.1343
Response Latency (ms)			
Size Class	0.0098	1	0.9218
Treatment	2.094	3	0.114
Size Class x Treatment	0.2511	3	0.8601
Burst Total Distance Traveled (mm)			
Size Class	0.4993	1	0.4834
Treatment	0.8665	3	0.4653
Size Class x Treatment	0.8662	3	0.4654
Burst Ave. Velocity (mm/s)			
Size Class	12.0209	1	**0.0011**
Treatment	0.0725	3	0.9744
Size Class x Treatment	1.6216	3	0.1973
Burst Duration (ms)			
Size Class	28.9175	1	**0.0001**
Treatment	2.915	3	**0.0442**
Size Class x Treatment	1.1802	3	0.3276
Max. Burst Velocity (mm/s)			
Size Class	2.4542	1	0.1241
Treatment	0.5334	3	0.6617
Size Class x Treatment	0.6336	3	0.5971

Based on the binomial density function, small juvenile kelpfish exhibited significantly greater mortality than expected based on the survivorship probability of large juvenile kelpfish at high gnathiid treatment levels (*p* = 0.003). However, kelpfish exhibited no significant difference in mortality between size categories at medium gnathiid treatment levels (*p* = 0.31), indicating that the number of gnathiids needed to cause mortality in juvenile giant kelpfish is dependent on fish body size (Table 2).

**Table 2:** (a) Counts of live and dead giant kelpfish for medium and high gnathiid treatments and (b) probability of survival of giant kelpfish for all gnathiid treatments.

a)						
Size Class	Medium Treatment			High Treatment		
	Alive	Dead	Total	Alive	Dead	Total
Small	7	1	8	6	7	13
Large	7	2	9	7	1	8

b)				
Size Class	Control	Low	Medium	High
Small	1.000	1.000	0.875	0.462
Large	1.000	1.000	0.778	0.875

## 4 Discussion

As consumers, potential disease vectors, and prey, parasites and micropredators are an important component of ecological food webs in aquatic systems (e.g., Thieltges et al., 2013; Orlofske et al., 2015; Wood and Johnson, 2015), including kelp forests (Morton et al., 2021). Because of their unusual life cycle, gnathiids are typically overlooked by marine ecologists, including parasite ecologists. Although our knowledge of gnathiid ecology comes mostly from studies in tropical coral reefs, the majority of temperate studies have been conducted in rocky intertidal (Smit et al., 2003; Welicky et al., 2018) and estuarine habitats (Hayashi et al., 2020). Our study here is one of the few ecological studies of temperate gnathiids in sub-tidal kelp forests (Hobson and Chess, 1976; Hammer, 1981; Stepien and Brusca, 1985; Morton et al., 2021).

The gnathiid collected in this study conforms to the description of *Gnathia tridens* (Menzies and Barnard, 1959), first collected in kelp habitat off Pt. Conception, California (34.4486° N, 120.4716° W). The species was described based on a single male specimen collected from a bottom-grab, and thus no host data were available. The host fish species used in this study is a confamilial (Family Clinidae) of species that are common hosts of gnathiids in temperate intertidal systems in South Africa (Smit et al., 2003). Thus, our collection of this species off San Diego and from the giant kelpfish constitutes both the first known host and a range extension, and suggests that clinid fishes are common hosts of gnathiids in temperate systems.

### 4.1 Comparison of trap types

Our comparison of multiple trap designs based on a comparison of modifications of emergence traps (Artim and Sikkel, 2016) similarly revealed that the addition of a light source and a live fish as “bait” increased the number of gnathiids collected. Standard emergence traps rely on “accidental” entry into the funnel at the top of the trap, whereas lights and live fish offer sensory ques that directly attract gnathiids to the trap. In addition to informing trap design for the remainder of the study, these findings confirm that, as with tropical gnathiids (Jones et al., 2007; Artim and Sikkel, 2016; 2020), at least some temperate gnathiids are attracted to artificial light and that giant kelpfish is at least one host for *Gnathia tridens.*

### 4.2 Environmental predictors of abundance

Soft-bottom embayments and rocky reefs form extensive habitats within southern California marine ecosystems. Bays include both unconsolidated sediments and seagrass beds, which provide structural relief and habitat for many fishes. The deep areas of these bays often provide an extension to coastal habitats and can create seasonal variation in fish assemblages (Froeschke and Allen, 2006). Seagrass beds not only provide a variety of critical ecosystems services such as stabilizing sediment and promoting biodiversity (Fonseca and Cahalan, 1992), but they also provide food to epifaunal invertebrates and fishes, along with physical refuge (Lewis and Anderson, 2012). From our initial trapping study, gnathiids rarely inhabited embayments, which could be attributable to multiple factors. First, bay habitats could be host-limited. Although at least some gnathiids can feed on soft-bodied invertebrates (Nicholson et al., 2020), fishes are their main hosts, and large-bodied fishes provide the easiest “targets”. Studies in coral reefs have shown that although gnathiids do occur in seagrass beds adjacent to reef habitat (Santos and Sikkel, 2019), they are less abundant in seagrass beds where fish biomass is lower (Sikkel et al., 2019; Artim et al., 2020) and fish biomass is related to gnathiid abundance in reef habitat. Unlike seagrass beds that are adjacent to tropical reefs and are visited by reef-associated fishes, the bay habitats sampled here are isolated from rocky reefs. Thus, a paucity of large-bodied fish hosts may be a limiting resource in bay habitats. In contrast, kelp forests have a high density of fishes that include adults of several large-bodied species. In addition, although gnathiids feed on fish blood, planktivorous and microcarnivorous fishes are also known to eat gnathiids (Artim et al., 2016). Thus, another possibility is that bay habitats include sufficient numbers of potential gnathiid predators, along with fewer hiding places for gnathiids due to finer sediment and the lack of structurally complex rocky substrata.

Among the abiotic factors included in this study that could influence gnathiid density, lunar period (measured by the percentage of lunar illumination at the time a sample of gnathiids was collected) was a significant predictor. In particular, higher numbers of gnathiids were collected in emergence traps during full illumination periods (full moons or 100% illumination). Lunar phase shifts have the ability to change the distribution, abundance, and activity patterns of many marine organisms (Benoit-Bird et al., 2009; Seymour et al., 2018; Owen et al., 2019). However, only a few studies have examined how lunar period may impact gnathiid activity. Jacoby and Greenwood (1988) found that gnathiids at Heron Island, Australia, emerged from reefs in higher densities at full moon. They posited that gnathiids emerge during the full moon lunar phase because they are able to find fish hosts more easily. However, other studies have found mixed results. For example, Grutter et al. (2011) found that emergence rates of gnathiids varied among lunar phases, being lowest around new moon, and Welicky et al. (2013) found no consistent effect of lunar cycle on gnathiid activity in the Caribbean. Differences among studies could be due to differences in location, which may determine gnathiid species composition as well as local current patterns associated with the lunar phase and other factors. For example, tidal currents are very weak at the sites studied by Welicky et al. (2013) and the studies also differed in trapping techniques, with studies on the Great Barrier Reef using only emergence traps whereas those in the Caribbean (e.g., Welicky et al., 2013) included cages “baited” with live fish. To understand the impact of lunar cycles on gnathiid activity, sampling over multiple spatial scales involving a variety of trap designs is necessary.

Wave height was another indicator of the number of gnathiids collected in traps. Greater wave activity (greater wave height) was associated with a decreased number of gnathiids collected in our emergence traps. Gnathiids may be unwilling to forage during large wave events because high wave action could reduce their swimming and attachment ability. Nicholson et al. (2019) found that gnathiids occurred in highest numbers at or below 1 m above the reef substratum and were collected in lower densities 2–3 m above it. Gnathiids can swim into the water column to exploit mobile fish, but they may be limited in how far they are able to swim, especially in turbulent conditions. Turbulence may also decrease their ability to use chemical cues, which are also important in host-finding (Vondriska et al., 2020). With the exception of one study of a temperate rocky intertidal gnathiid (Welicky et al., 2018), this study of gnathiid activity is the only one conducted in high wave energy systems. It is worth noting that the densities of gnathiids we collected during calmer wave energy days were comparable to the densities collected in studies using similar techniques in low wave-energy environments in the Great Barrier Reef (Jones and Grutter, 2007; Paula et al., 2021), and the Caribbean (Artim et al., 2020), further suggesting that wave activity may be an important determinant of gnathiid activity. In addition to swimming and host-finding, large waves may also impact gnathiids through increased suspended sediments in the water column that may smother refuges used for breeding and moulting (Alldredge and King, 1980; Balata et al., 2007), in addition to decreasing light attenuation in the water column (Connell, 2005).

In coral reef systems, substrata have a strong impact on gnathiid distribution and abundance. In particular, dead coral, rubble, and sand appear to be suitable habitat for gnathiids, whereas live coral does not (Artim and Sikkel, 2013; Santos and Sikkel, 2019; Artim et al., 2020; Paula et al., 2021). In this study, we compared gnathiid densities in rocky and sandy substrata and found no difference between them. This suggests that the coarse sand found in kelp forest habitats is suitable for gnathiids, and that a substantial amount of rocky substratum may not include sessile predators of gnathiids (e.g., cnidarian polyps). However, we did not incorporate variation in the composition of rocky reef substratum, so that finer-scale analysis of substrata may reveal other predictors of gnathiid distribution within rocky reefs.

### 4.3 Gnathiid impacts on host fish

We chose to explore the lethal and sub-lethal impacts of zuphea stage gnathiid isopods using juvenile giant kelpfish in an effort to assess the potential impacts of gnathiids on successful recruitment of juvenile fishes to adult populations in temperate systems. There was a significant difference in the probability of survival for smaller compared to larger fish at the highest levels of gnathiid infestation, indicating that parasite-induced mortality is greater for smaller fish. Our findings therefore further illustrate the importance of gnathiids as a potential cause of mortality in smaller, more recently settled fishes as seen in the tropics. A single gnathiid is sufficient to kill a French grunt (*Haemulon flavolineatum*) at 7–15 mm SL, (Artim et al., 2015), and two damselfish (*Acanthochromis polyacanthus and Neopomacentrus azysron*) < 13 mm exposed to 1–3 gnathiids experienced mortality 16% of the time compared to larger individuals that experienced no mortality (Grutter et al., 2008; Penfold et al., 2008). However, the relationships between gnathiid infestation, host body size, and mortality varies among fish species (Sellers et al., 2019). Giant kelpfish are larger at settlement (ca. 25–30 mm; T.W. Anderson, pers. obs.) and therefore are larger as juveniles than recruits of many other tropical fishes. Therefore, we scaled up the number of gnathiids to account for this size difference. Given that globally this relationship has been established for so few species, mostly from one family, more studies are clearly needed to understand the underlying causes of this variation.

For fish that survive gnathiid infestation, both burst and ambient swimming behaviors can be impacted. Impaired escape and other behavior as a response to parasitism has been observed in a range of animals (Poulin, 1995). For example, parasite intensity causes changes in infected lizards by altering running or leaping to avoid or flee from predators (Garrido et al., 2015), which in turn, causes infected individuals to have higher encounter rates with predators. Studies like this have shown that most parasitized hosts experience disproportionately greater predation (Genovart et al., 2010), and infections can increase energetic costs incurred by the host by increasing locomotion or through immune system functions (Grutter et al., 2011).

The ability to escape a predator and forage efficiently are key behaviors influencing the survival of fishes. Swimming speed and behavior are often used as measures of the performance capabilities of fish, as they are important for predator avoidance, foraging, or finding suitable habitat (Green and Fisher, 2004; Herbert and Steffensen 2005; Binning et al., 2014; Allan et al., 2020). Escape responses involve fast-start swimming, classified as a high-energy swimming burst, either from rest or from a steady-state swim, and are critical in the ability of mobile fish prey to escape their predators (Batty and Blaxter, 1992; Domenici and Blake, 1997; Walker et al., 2005). Prolonged or ambient swimming is used when fish forage, maintain territories, or move through habitats (Sfakiotakis et al., 1999). Both modes of swimming influence fish survival, either directly for successful predator evasion (Grutter et al., 2011) or indirectly through foraging success and finding suitable habitat (Welicky and Sikkel, 2015).

Although recent studies have reported decreased sustained swimming in parasitized fish (Grutter et al., 2011; Allan et al., 2020), we found that infested individuals performed equally well as uninfested conspecifics in almost all burst and ambient swimming metrics. Our findings are consistent with a similar study by Östlund-Nilsson et al. (2005), which examined the impact of *Anilocra* sp., a much larger temporary ectoparasite, on fish swimming performance in the cardinal fish *Cheliodipterus quinquelineatus*. The lack of an effect from gnathiid infestation on swimming performance overall was unexpected but may be due to variability in individual susceptibility. The susceptibility of individual fish to the same level of gnathiid infestation is not uniform among species; susceptibility may be driven by the scale thickness of a fish, scale type, cortisol response, or even mucus secretions that act as a protective cocoon (Nagel and Grutter, 2007; Sikkel et al., 2011, 2014; Coile and Sikkel, 2013). Most recently, Allan et al. (2020), found that a single gnathiid caused fast-start performance and swimming behavior to significantly decrease in recently settled *Pomacentrus amboinensis*. Similarly, Sellers et al. (2019) found that sub-lethal gnathiid loads negatively impacted the ability of juvenile *Stegastes leucostictus* to defend a territory against an uninfected conspecific of similar size.

Contrary to our initial predictions, the highest treatment level (nine gnathiids) for our fish swimming performance experiments did not differ from any of the other gnathiid treatments. This may be explained by the ability of impaired fish to compensate for swimming under strenuous circumstances (Poulin, 1995; Barber et al., 2000). It may be that there is an optimal host:parasite ratio, whereby the number of gnathiids needed to impair a host's swimming mechanics is a direct function of fish size and available blood volume, and above or below this ratio, fish may be able to compensate (Jones and Grutter, 2005; Artim et al., 2015). At lower gnathiid numbers, fish may not be impaired at all, and thus show no significant signs of infection. At the highest numbers of sub-lethal gnathiid infection, fish may only react to the most life threatening stimuli and engage in riskier behavior as a long-term energy-saving mechanism (Giles, 1987; Godin and Sproul, 1988).

Gnathiid isopods are found in all oceans from the intertidal to the abyss and have multiple potential impacts on ecological communities through their role as consumers, prey, and vectors (Sikkel and Welicky, 2019). Our understanding of their ecology is limited by their unusual life cycle, resulting in their being overlooked by both ecologists and parasitologists, and the small number of research groups who study them. We have provided data on habitat associations and one potential impact on juveniles of one host in a geographical area where no previous research on gnathiid ecology has been conducted. This paper sets the stage for a broader range of ecological studies on this species that include host range, diel activity patterns, consumers, and potential transmission of blood-borne parasites.

## Funding statement

Funding was provided by a California State University Grant, the American Academy of Underwater Sciences, the California State University Council on Ocean Affairs, Science and Technology, the American Museum of Natural History Lerner Gray Fund for Marine Research, the Western Society of Naturalists, and San Diego State University's Harold and June Grant Memorial Scholarship, Mabel Meyers Scholarship, and Elliot Family Fund Scholarship (to CAS). Additionally, initial training for this project was completed in St. John, USVI and was supported by NSF OCE-1536794 (to 10.13039/100015534PCS).

## Declaration of competing interest

None.
